# Dental Hints Department

**Published:** 1906-10

**Authors:** B. H. Teague

**Affiliations:** Aiken, S. C.


### DENTAL HINTS DEPARTMENT

Conducted by B. H. Teague, D. D. S., Aiken, S. C., to whom all contributions to this department should be sent.

Turpentine is better than oil for use when drilling hard steel, saw plate, etc.—*Popular Mechanics.*

A convenient and inexpensive holder for polishing gold crowns can be constructed out of the ordinary clothes-pin.—*Exchange.*

Extension for Prevention.—My advice is, cut back until you think you have cut enough; then cut some more for fear you haven't.—John Campbell, *The Dental Era.*

Coffee is a good antidote for acetanilide. This should be mentioned to patients when prescribing it, as it may prevent much harm, and save life.—Dr. J. A. Burnett, *Medical Brief.*

Separating Varnish.—Dissolve three ounces of borax in a quart of water, bring to the boiling degree, and add six ounces of powered shellac.—Dr. A. Doughaday, *Western Journal.*

Extracting Teeth.—Frequently teeth can be extracted absolutely without pain by the injection of a normal salt solution. We get the psychical effect and in addition quite a little anaethetic effect.—Dr. Arthur D. Black, *Review.*

A small amount of acetanilide put in the hollow of an aching tooth will quickly relieve it. Guaiacol is superior to acetanilide for toothache, and should always be used in place of acetanilide, when on hand.—Dr. J. A. Burnett, *Medical Brief.*

Cementing Inlays.—To maintain pressure on inlays in proximal cavities until the cement crystallizes, spring a piece of nursing bottle tubing between the teeth. Allow it to remain until the cement has set.—Oliver Martin, *Review.*

To Polish a Porcelian Crown or any Porcelian Tooth that has been Ground.—Make a very soft paste by mixing spirits of turpentine, gum camphor, and pumice flour. Keep your polishing wheel wet with this mixture while repolishing the tooth.—W. H. Spaulding, *Dental Summary.*

Twisting Ligatures.—Wire ligatures should first be drawn tight with the hands and then twisted. If the attempt is made to tighten by twisting only, the wire will break before any tension to speak of is attained.—*Western Dental Journal.*

It should not be forgotten that the porcelian art in dentistry is the art that conceals art, and the cultured and esthetic are going to have it, and more and more so as the years go on.—Dr. R. B. Tuller, *American Dental Journal.*

Care of the Mouth.—Two drops of camphor on your tooth brush will give your mouth its freshest, cleanest feeling imaginable, will make your gums rosy, and absolutely prevent anything like cold-roses or affections of the tongue.—*Dental Digest.*

Obtunding Sensitive Dentin.—Moisten the cavity freely and place in it a bit of solid sodium dioxide, allowing a drop of water to fall upon it. A brief moment of pain follows, after which the excavation may be proceeded with almost painlessly. For deeper work a second application may be necessary; usually with no pain at all.—E. M. Soule, *Items of Interest.*

Anterior Teeth Should not Occlude.—Still other dentists use the shoulder for the lower fronts to meet, a very unwise thing to do, as two serious conditions result, nafely, displacement of the plate from the rear, and constant absorption of bone from pressure, resulting in a flabby gum.—L. P. Haskell.

To Preserve Rubber.—One part of ammonia in ten to twelve parts of water will preserve soft rubber. Dip rubber pipes, etc., in a glass jar filled with this solution. For the ammonia bottle use a rubber stopper; it is better than a glass one. —*Cosmos.*

Spray the Gum.—Did you ever spray ethyl chlorid on the gums around the root of a tooth that you were preparing for a crown? Try it and your patient will repay you for your trouble.—*Amer. Dental Journal.*

Pyorrhea Alveolaris.—A line of "continuous, mild medication" which has proven efficacious consists in placing silver bands about the roots of the teeth, to furnish silver salts for their typical effect.—W. V. B. Ames, *Dental Review.*

His plate-glass door having been smashed, a German dentist has advertised thus: "If the person who damaged my premises will report himself I hereby agree to pull out a few of his teeth for nothing." This seems to be a case of "turning the other's cheek."—*The Tatler.*

Adrenalin-Chloride.—The application of adrenalin-chloride to freshly exposed or healthy pulps is a decided success, but I have found from unpleasant experience that its application must be confined to such cases. The slighest pathological change in the pulp tissues seriously interferes with its successful use.—I. N. Taylor, *British Dental Journal.*

Filling Material for Deciduous Teeth.—I believe there is no filling material for deciduous teeth as satisfactory as base-plate gutta-percha, especially in approximal cavities. It works as a twofold blessing, closing the cavity and procuring the space required for the insertion of the permanent filling at a future time.—R. M. Pearce, *Dental Review.*

Treating Cavities With Nitrate of Silver.—I believe it is a good practice when you have some evidence of a superficial decay to touch it with nitrate of silver and see how deep it will go. In a few weeks that will show you how far the area of decay has gone. Lots of times you need not go any further, as that will stop the decay.—J. N. Crouse, *Dental Digest.*

To Prepare the Floor of the Cap for a Porclain Crown.—Puncture the floor of the cap from the root side with a pointed three-cornered instrument. A puncture thus made turns up a burr on the porcelain side, which becomes firmly imbedded in the body of the piece, and is fully as retentive as a headed pin in the facing.—L. A. Arnold, *The Dental Surgeon.*

Income of Medical Men.—The income of medical men depends on their fees. In New York an authority on the subject gives the following estimate: Two or three physicians make over $100,000 a year; four or five range from $50,000 to $60,000; fifty from $25,000 to $30,000; 150 from $10,000

to $12,000; about 300 from $5,000 to $6,000; 1,500 from $2,000 to $3,000, and the remainder from $300 to $1,000.—*Newspaper.*

To Stop Puncture in a Rubber Dam.—For punctures of the dam or to stop leakage around the lower anterior teeth, dry the dam with cotton, melt a little baseplate wax on a spatula and carry to points of leakage. This result will gratify you.—A. C. Peterson Elmwood, Ill., *in Tri-State Quarterly.*

San Francisco Dentists.—The dental profession of San Francisco appears to have been hard bit by the recent earthquake there. It is reported that 500 dentists at least have had their offices destroyed, three dental schools have been completely wrecked, and in addition, seven dental depots abolished. —*The Dental Surgeon.*

Treatment for Canker Sore Mouth.—I have found the full strength aromatic sulphuric acid almost a specific for this condition. I prescribe internally tincture of ferric chloride gtt. v.; potassium chlorate gr. iii.; water ½ oz. every three hours in lemonade.—J. E. Power, *The Tri-State Dental Journal.*

Root Treatment.—"Next time you want to get a liquid medicament to the end of a root canal do it this way: Flood the canals with the liquid, introduce a Downie broach and revolve it the wrong way; the blades of the broach will operate like the screw propeller on a steamboat. Let me know what you think of the idea."

It's "all right."—*Office and Laboratory.*

Method of Removing an Unbaked Inlay from the Cavity.—In a recent operation where I used Roach's Opaque Dental Porcelain to a posterior tooth, I lessened the difficulty of removing the inlay from the cavity by imbedding in the center of the unbaked inlay while in the cavity, a small and fine piece of platinum wire, which I used as a handle to remove it by. When the inlay was baked and cemented to the cavity, I cut off the wire with a sharp steel instrument, the wire being so fine that it did not show in the inlay.—E. M. Fernandez.

To Cleanse Mercury.—Put a 10 per cent solution of nitric acid in an iron ladle and add the mercury. Place the ladle over a blacksmith's forge, says Machinery, until the nitric acid boils. The dirt will rise to the top and the mercury, perfectly clean, remain at the bottom. Do not let the mercury boil, the fumes are poisonous.—*Popular Mechanics.*

HYGIENIC FILLINGS.—The sealing of the tubuli is the one important feature in the preservation of tooth structure. We now have a material for this purpose that is better than cement; material that is ready for instant use, that requires no mixing, that sets instantly, that is non-conducting, adhesive, and antiseptic, thus forming the ideal lining; it firmly anchors the first piece of gold, upon which the rest of the filling may be packed without danger of loosening or dislodgment. This material is gold anchor intermediary, composed of thymol, dammar, baric sulphate, and oil of cloves.—ALISON R. LAWSHE, *Items of Interest.*

THE GOLD MATRIXS PROTECTION FROM FUSING.—It is not necessary that any matrix be invested; when gold is used it is protected from fusing by coating it, preferably with rouge because of its great fineness and affinity for a smooth surface. The rouge is bought in powder and spatulated with alcohol and water. It will resist heat to an astonishing degree.—W. A. CAPON, *Items of Interest.*

A HOT BRICK.—A common brick is the best thing to warm rubber on before packing. It holds heat a long time and the rubber *will not stick* to it. Don't get a smooth one, just a common red brick, and you will wonder why such a good thing wasn't found out before..—O. F. BRIGHAM, *American Dental Journal.*

ANCHOR BANDS.—There is no question whatever of the superiority of clamp bands for anchorage upon the molar teeth; they fit better, are more easily applied, are far more stable, and may be easily removed at any time. They may be easily cemented in place, overcoming the last objection to their use. The old-fashioned plain band, simply cemented on to provide anchorage for the appliance, has served its time, and better things have taken its place.—*Western Dental Journal.*

STERILING ROOT CANALS.—In from ten to fifteen minutes the electrolytic action of a constant galvanic current of from one to two milliampéres suffices for sterilizing completely root canals inaccessible to instrumentation. Injecting a 75

per cent aqueous solution of common salt, thoroughly saturating the canal contents, gives a good conductor. If the anode or positive pole is made of platinum, it will deposit chlorine, oxygen, and hydrochloric acid, these ions promising a decided anti-bacterial effect.—Dr. Kurl Hoffendahl, *Dental Cosmos.*

Liquid Flux.—The best liquid flux that I have ever used, which I compound myself, and find ever ready and faithful, is:

| | | | |
|---|---|---|---|
| R. | Boracic acid | | |
| | Borax .. .. .. .. | āā | ℥ ss. |
| | Ammonia muriate .. .. .. | | grs. xii. |
| | Pot. carb. .. .. .. .. | | grs. v. |
| | Aqua dist., q. s. .. .. .. | | ℥ iv. |

It will keep indefinitely, and can be used on any metal for hard soldering. It should not be used, however, in connection with porcelain.—H. E. Davis, in *Dentist's Magazine.*

Porcela.—I have found that a cleansing preparation called Porcela, recently placed upon the market, is of considerable value to the dentist. This powder is especially compounded for the purpose of cleaning and polishing bathtubs and other porcelain fixtures. Moistened with water and used with a felt wheel or the lathe it will impart an excellent polish to porcelain teeth which have been ground.—Dr. Edward C. Duryee, *Cosmos.*

Bending the Arch.—The expansion arch often produces too great outward movement of the molar teeth, due to too much spring upon the molars, and too little space between the bicuspid teeth and the arch. Expand the anterior portion of the dental arch first, ligating the bicuspids (and cuspids if necessary), very tightly to the arch, and making little effort to expand in the molar region until towards the last of the operation. The fact that the expansion arch sometimes displaces the molars is due largely to failure to observe the foregoing details.—*Dental Register.*

Prescription Writing—Latin v. English.—Apropos of recent correspondence concerning medical prescriptions, whether they should be written in Latin or plain English, our contemporary *The Globe,* tells the story of a physician who, prescribing syrup of buckthorn for an old lady, wrote it in the usual form, "Syr. Ram. Cat." When she called again, he asked her whether she had taken the medicine. "No," she replied, indignantly, "I ain't a goin' to take ram cats for anybody."—*The Dental Surgeon.*

Blind Abscesses.—Quite frequently in blind abscess cases, after the pus formation has been checked, we have a weeping of serum from the canals. I find this condition more often associated with the superior laterals than with any other teeth, although it is sometimes found in connection with all teeth from which abscesses have been treated. An excellent remedy to use here is eucalyptol, to which thymol has been added in the following proportion:—

| | | | |
|---|---|---|---|
| R. | Eucalyptol | .. .. .. .. | f ʒj. |
| | Thymol | .. .. .. .. | gr. x. |
| M. | Sig.: | Use as directed. | |

If this remedy fails to check the secretion and the liquid is *serum* (not pus), no hesitancy need be had in filling the root, although the canals should be moist.—Dr. J. P. Buckley, *Review.*

Colored Arsenic Mixtures.—Dr. G. V. Black long ago suggested that we mix our arsenic with sufficient lampblack, so that when placed within the tooth it may be seen plainly where it lies, and we may be able to detect any particles that might be disposed to crawl out of the cavity, anl also in order to insure a complete removal of the dressing after the pulp has been devitalized. Anyone who will take the trouble to put up, or order from the druggist, a mixture of arsenic, morphin, and cocain, with lampblack in sufficient quantity to make a dull black, and will use this mixture for a time, would never think of doing without it.—Garrett Newkirk, *Dental Review.*

Inlay.s the Gold Matrix.—It has been suggested that the matrix be formed of No. 36 gold plate, and that the portion which comes in contact with the margins, and for a considerable distance below, be left upon the inlay. After the cement is thoroughly hardened the surplus gold that overhangs the margins is to be cut off and the remaining gold burnished to cover up the line of cement. The advantages are that the coating is so heavy that it will not pull in fusing the porcelain, and it makes a more perfect union between the inlay and the tooth. I have found this method of great value.—F. T. Van Woert, *Dental Cosmos.*

Formaldehyde.—Polymerized formaldehyde—formaldehyde in a solid form—has all the qualification necessary to a perfect dental antiseptic. It is most penetrating, it is soluble in water; it does not produce a coagulum; when properly applied its toxic or escharotic effect is *nil* and pain seldom or never follows its application unless it be in dead teeth, in which the simple act of opening seems to arouse the latent forces around the apical portion of the tooth. But the pain and soreness following its application invariably subside within a few hours, when the tooth is ready for permanent filling.—L. B. Lawrence, *Dental Cosmos.*

Indications for use of Mouldable Porcelain.—Mouldable dental porcelain, on account of its opacity, is not indicated in the exposed cavities in front teeth. It is indicated in large cavities in any tooth posterior to the cuspid. In some mouths, however, where the teeth are very conspicuous, its use in the bicuspids is not permissible. There is a class of cavities in molars which the operator can not fill with gold. He has the alternative of crowning or filling with amalgam. The latter but postpones the former, and the crown is not desirable for several reasons. Mouldable porcelain is most especially indicated here, and is probably its largest field of usefulness at the present time.—Dr. E. G. Perry, *Review.*

Oil of Cajeput.—Dr. Arthur Matteson, many years ago, told us about the use of this agent in connection with gutta-percha. In placing a temporary stopping in a shallow or saucer-shaped cavity, if the latter be moistened with oil of cajeput warm gutta-percht will adhere to it with special tenacity. It is also as excellent root canal dressing previous to filling with the gutta-percha cone—better for either of these purposes, I think, than the oil of Ecalyptus, and possesed of equal antiseptic properties.—Garrett Newkirk, *Passadena, Cal.*

Amalgam Fillings.—The best method of making amalgam fillings tight is to use all the force we can and pack it along the walls with a very small instrument. The amalgam must be hard enough. It is not so difficult to make walls that are practically perfect. It is easy to make walls that will not show spaces with the naked eye, but it is not so easy to make walls that will not show spaces with the microscope. Still we can make fillings that will do our patients much good, often where a gold filling would not answer as well.—G. V. Black, *Chicago.*

Nitrate of Silver.—For ten years I have made it a rule to see that all the teeth were given a good treatment with a saturated solution of nitrate of silver, as soon as possible after eruption. I simply dry off the surfaces and put on the solution with a small swab, letting it stay a minute, during which time I push it with an explorer down into the sulci. I give this as an invariable rule, carried out with great success for the above length of time. Decay is generally prevented, or when it does occur is greatly delayed.—Harry F. Hamilton, *International Dental Journal.*

Treatment of Pyorrhea.—First inject into the pockets a few drops of a 15 per cent solution of cocaine until the tissues are anesthetized; then with a set of Young's pyorrhea instruments, as revised by Dr. Good, I go up, if necessary, to the end of the roots, being careful to remove perfectly all of the calcareous deposits at the first sitting, and when I am satisfied there is nothing left I polish carefully and inject into the pockets a few drops of pure lactic acid two or three times a week until results are produced, which are slow in some cases, but always sure. I then instruct the patient carefully as to the correct way to brush the teeth and gums. To say the least of it, I believe and hope I am effecting cures along this line.—Dr. J. H. Nicholson, *Era.*

Failure of Fillings.—There are many things that enter into the question of failure of fillings. Imperfect preparation of cavities is by far the largest factor in the problem. The dentist who practices extension for prevention, either in its extreme form or with modifications to suit the cases, can trace the recurrence of decay to faulty preparation. Possibly in order to avoid a display of gold or to save time—to save pain to the patient, or from laziness and a desire to do the easy thing rather than the best, a compromise was made. A compromise in most things of life only postpones the day of reckoning, and this is especially true in the preparation of cavities. —W. A. Hoover, *Review.*

Cocaine and Adrenalin-Cocaine Anaesthesia.—A long and careful series of experiments by Sikemeier shows that contrary to some recent teachings, adrenalin, injected with or even before cocaine, does not lessen its toxic action. Similar doses with and without adrenalin had the same effect, and the fatal dose was the same also. The effect of the cocaine was not at all increased by the addition of adrenalin, and just as much cocaine was required if adrenalin was added as without it. The anæmia produced by the adrenalin is, however, a great advantage, and it should always be used when hemorrhage is feared. The increase of medullary anæsthesia by the addition of adrenalin is due to the anæmia of the cord, which in itself produces anæmia of the limbs. Zeigon showed that adrenalin alone, injected beneath the dura, produced this effect.—*Therapeutic Gazette.*

To Remove Model from Articulator.—It not infrequently happens that a model is broken in removing it from the articulator. To ensure the easy removal of even a very thin model, have three or four sizes of iron plates convenient. These plates are one-twelfth of an inch in thickness, and about the size of the bottom of the model. The plate should have three or four quarter inch holes in it. In putting a model on the articulator, put the plate on the jaw of the articulator and the model on the plate with a little soft plaster to hold the plate to the articulator, and the model to the plate. When it is desired to remove the model, slip a knife or chisel between the plate and articulator, and prize the plate and model off together. As the plate is rigid there is no danger of breaking the model. The plate is flasked with the model and recovered after vulcanising.—C. R. Smithers, *Western Dental Journal.*

Effects of Abscess Arising from Temporary Teeth.—I have thought for a long time that abscesses and septic troubles of temporary teeth ought to have some considerable effect on the teeth of succession, but I have been unable to prove it until lately. At St. Thomas's Hospital I have had to do all the extractions myself, and I have come across quite a number of cases in which the succeeding teeth have been spoilt by an abscess of the temporary teeth. Here is a model in which the second bicuspid appears to be erupting outside the second temporory molar in a child, aged 8½. The bicuspid is necrosed and cast off; it was dead and septic when I took it out, and there was a chonic abscess of the temporary molar.—*Exchange.*

Be Careful With Your Anaesthetic.—The universal dread of pain and the necessity for the infliction of more or less pain by the dentist in his operations, has caused untold suffering to the human race and the loss of thousands of teeth. We and our patients sometimes think that if the element of pain could be eliminated from our operations, life would be a continual round of pleasure. Since this is true, it is small wonder that the use of anæsthetics is being pushed to the limit. Dentists are human and if they can substitute smiles for groans and compliments for execrations, the temptation is great to overstep the bonds of caution. Recognizing the above as universal traits of human nature, it requires courage to refuse an anæsthetic. Remember, however, it is a chance and responsibility which no one can divide with you. Can you afford it?

Reface Gum-Sections.—The ground faces of the surfaces that make the joints of a set of some makes of gum teeth are porous, and because of this condition, whether worn for a week or a year, drink in saliva with matter held in solution and suspension, with the result that if the teeth are reset without further treatment this organic material is carbonized during vulcanization, giving the joints of the remounted set an appearance as offensive as if rubber-filled. Chemical treatment of the teeth when taken from the old plate helps matters somewhat; but the way of ways is to reface the ground surfaces by means of a fine wheel—corundum, carborundum or even a fine sand-paper disk. This treatment removes all of the affected and infected material, and yet not enough of the porcelain to make any appreciable difference in the spaces between the sections.—Dr. W. H. Ellis, *Dental Magazine.*

